# Proprioceptive Control of Muscle Activation in Aging: Implications for Balance and Fall Risk

**DOI:** 10.3390/biology14060703

**Published:** 2025-06-16

**Authors:** Łukasz Oleksy, Anna Mika, Martyna Sopa, Artur Stolarczyk, Olga Adamska, Joanna Zyznawska, Rafał Buryta, Paulina Ciepiela, Jarosław Witkowski, Renata Kielnar

**Affiliations:** 1 Department of Orthopedics, Traumatology and Hand Surgery, Faculty of Medicine, Wroclaw Medical University, 50-556 Wroclaw, Poland; loleksy@oleksy-fizjoterapia.pl; 2 Department of Physiotherapy, Faculty of Health Sciences, Jagiellonian University Medical College Kraków, 30-688 Kraków, Poland; joanna.zyznawska@uj.edu.pl; 3 Oleksy Medical & Sport Sciences, 37-100 Łańcut, Poland; paulina.ciepiela1006@gmail.com; 4 Institute of Clinical Rehabilitation, University of Physical Culture in Kraków, 31-571 Kraków, Poland; 5 Institute of Applied Mechanics, Faculty of Mechanical Engineering, Poznan University of Technology, 61-138 Poznań, Poland; martyna.sopa@put.poznan.pl; 6 Department of Orthopaedics and Rehabilitation, Medical and Dentistry Faculty, Medical University of Warsaw, 02-091 Warsaw, Poland; artur.stolarczyk@wum.edu.pl; 7 Department of Ophthalmology, Faculty of Medicine, Collegium Medicum Cardinal Stefan Wyszyński University in Warsaw, 01-815 Warsaw, Poland; o.adamska@uksw.edu.pl; 8 Institute of Physical Culture Sciences, University of Szczecin, 71-065 Szczecin, Poland; rafal.buryta@usz.edu.pl; 9 Division of Sports Medicine, Department of Orthopaedics, Traumatology and Hand Surgery, Faculty of Medicine, Wroclaw Medical University, 50-556 Wroclaw, Poland; jaroslaw.witkowski@umw.edu.pl; 10 Faculty of Health Sciences and Psychology, Collegium Medicum, Institute of Physiotherapy, University of Rzeszów, 35-315 Rzeszów, Poland; kielnarrenata@o2.pl

**Keywords:** bioelectrical activity (EMG), proprioception, elderly adults, balance, fall risk

## Abstract

(1) Background: This study aimed to assess whether older adults exhibit greater discrepancies between intended and actual motor unit recruitment, which could affect the quality of muscle activation and potentially increase the risk of falls. (2) Methods: Forty-eight physically active older women were assessed (65 ± 6 years, 164 ± 6 cm, and 76 ± 7 kg). The bioelectrical activity (EMG) of the vastus lateralis oblique (VLO) and vastus medialis oblique (VMO) muscles were assessed during isometric testing with the knee joint bent to 75 degrees. The participants were instructed to press against a stable bar for 5 s at a specific percentage of their perceived force level (at 15%, 30%, and 60% of MVC) when the EMG activity was recorded. Balance was assessed using a stabilometric platform in a standing position. (3) Results: In all three thresholds, the bioelectrical activity of the VLO and VMO muscles significantly deviated from what was expected under the assumption of a nearly linear relationship between muscle force and bioelectrical activity. In each of the three thresholds, it did not exceed 10% MVC and significantly differed only between the 15% and 60% MVC thresholds. No significant differences were found between the dominant and non-dominant sides. A significant relationship was observed between the sway area (Area 95%) and the activity of the non-dominant limb VLO muscle. (4) Conclusions: Our results suggest that older adults experience deficits in muscle activation perception, leading to discrepancies between intended and actual muscle engagement, which may affect functional task performance and potentially increase fall risk.

## Simple Summary

This study assesses the actual recruitment of knee extensor muscle motor units at specific maximal voluntary contraction (MVC) thresholds in older adults and explores the divergence between perceived effort and actual muscle activation. We hypothesize that older adults may exhibit greater discrepancies between intended and actual motor unit recruitment, which could affect the quality of muscle activation and potentially increase the risk of falls. Forty-eight physically active older women participated in the study (65 ± 6 years, 164 ± 6 cm, and 76 ± 7 kg). The bioelectrical activity (EMG) of the vastus lateralis oblique (VLO) and vastus medialis oblique (VMO) muscles were assessed during isometric testing with the knee joint bent to 75 degrees. The participants were instructed to press against a stable bar for 5 s at a specific percentage of their perceived force level (at 15%, 30%, and 60% of MVC) when the EMG activity was recorded. Proprioceptive deficits in older adults may contribute to impaired motor control and reduced stability. Our results suggest that older adults experience deficits in muscle activation perception, leading to discrepancies between intended and actual muscle engagement, which may affect functional task performance and potentially increase fall risk. Future research should explore the effectiveness of targeted interventions on improving force control across different thresholds, ultimately aiding in fall prevention and enhancing functional independence in aging populations.

## 1. Introduction

Proprioception, defined as the body’s ability to sense position, movement, and force in space, is essential for maintaining balance, coordination, and postural control [1,2]. This sensory function is mediated by mechanoreceptors found in muscles, tendons, ligaments, and joint capsules, which transmit sensory feedback to the central nervous system (CNS). The CNS integrates proprioceptive input with other sensory signals, coordinating movement and maintaining stability in both static and dynamic activities [1]. In aging populations, proprioceptive function declines due to changes in both the peripheral and central nervous systems, significantly impacting motor performance, balance, and mobility [1,3].

It has been reported that age-related proprioceptive decline is primarily attributed to reduced muscle spindle sensitivity, axonal atrophy, and neurochemical changes in the brain [3,4]. These factors impair neuromuscular control, alter joint biomechanics, and reduce the speed and accuracy of movement planning and execution [5]. As a result, older adults often struggle to maintain stability during both static posture and dynamic activities, increasing their susceptibility to falls and associated injuries [1,4]. Falls represent a major public health concern among older adults, with approximately one in three individuals over the age of 65 experiencing a fall each year [6]. Among these incidents, up to 20–30% result in moderate to severe injuries, such as hip fractures, head trauma, or lacerations, which significantly reduce mobility, independence, and overall quality of life [7]. Notably, hip fractures are particularly devastating, with mortality rates reaching 20–30% within one year post-injury [3]. Furthermore, more than 50% of elderly individuals who sustain a serious fall-related injury are unable to return to independent living [6,7,8]. These statistics underscore the urgent need for a better understanding of the neuromuscular and proprioceptive factors contributing to fall risk, particularly as the global aging population continues to rise. Our study contributes to this growing body of knowledge by exploring how proprioceptive control of muscle activation is compromised with age, ultimately affecting balance and stability.

In addition to these age-related changes in proprioception, muscle function also deteriorates. Older adults may still generate sufficient force for motor tasks but often experience deficits in force control, particularly during submaximal efforts [4,5,9]. Franco et al. [2] reported that fluctuations in muscle force increase motor output variability, particularly during low-intensity contractions (2–10% maximal voluntary contraction, or MVC). This suggests that older adults may struggle to maintain intended force levels and limb trajectories. Studies on electromyographic (EMG) activity during daily activities indicate that older adults, particularly those with reduced knee extensor strength, show greater muscle engagement in tasks like standing up, climbing stairs, and rising from a chair [10]. This increased muscle activity is often compensatory as weaker muscles are required to perform tasks demanding a higher force output, further increasing variability in muscle activation and reducing efficiency [2,11].

Additionally, older adults appear to exhibit greater variability in controlling perceived force levels regardless of contraction intensity [12]. This increased variability, particularly at lower force levels, has been observed by some authors [13,14]. However, when higher force demands (e.g., 20% of MVC) are used, errors in force perception are reduced in older adults compared with younger adults. This suggests that lower levels of force (e.g., 10% MVC) may be particularly difficult for both age groups to control [14,15].

Our study is the first to assess the recruitment of knee extensor muscle motor units at specific MVC thresholds in older adults and to explore the divergence between perceived effort and actual muscle activation. Although previous studies have examined age-related variability in force production and EMG activity [2,12,13,14], our study uniquely investigates the extent of divergence between perceived effort and actual EMG-based muscle activation across multiple submaximal thresholds (15%, 30%, and 60% MVC) in older adults performing knee extensions without visual feedback. This approach allows us to isolate proprioceptive contributions to motor unit recruitment and explore the sensory–motor mismatch under controlled conditions, providing novel insights into neuromuscular control in aging populations. We hypothesize that older adults may exhibit greater discrepancies between intended and actual motor unit recruitment, affecting muscle activation quality and potentially increasing fall risk. Additionally, we propose that these discrepancies may be more pronounced in tasks performed by the non-dominant side of the body. Based on these findings, it may be possible to suggest the most appropriate type of training or rehabilitation for fall prevention in older adults.

This study aimed to assess whether older adults exhibit greater discrepancies between intended and actual motor unit recruitment, which could affect the quality of muscle activation and potentially increase the risk of falls.

## 2. Materials and Methods

### 2.1. Study Participants

Forty-eight healthy, physically active older women participated in the study (65 ± 6 years, 164 ± 6 cm, and 76 ± 7 kg). Participants with a history of neurological or orthopedic disorders in the past year were excluded.

Leg dominance was evaluated using the Step Test. Participants were fully informed about the research protocol and provided their written informed consent to participate in the study. They were familiarized with the study procedures prior to the measurements. Both knee joints were tested, and all measurements were conducted by the same examiners.

The study was approved by the Ethical Committee of the Regional Medical Chamber in Kraków (76/KBL/OIL/2014). All procedures were performed in accordance with the 1964 Declaration of Helsinki and its later amendments.

### 2.2. Study Design

#### 2.2.1. Bioelectrical Activity Assessment of the Muscles

Electromyographic (EMG) activity of the vastus lateralis oblique (VLO) and vastus medialis oblique (VMO) muscles was recorded during isometric testing with the knee joint at 75° using a TeleMyo G2 system (Noraxon USA, Inc., Scottsdale, AZ, USA), following the Surface Electromyography for the Non-Invasive Assessment of Muscles (SENIAM) guidelines [16]. Prior to electrode placement, the skin was cleaned and degreased with alcohol. Surface electrodes (Ag/AgCl) (Sorimex, Toruń, Poland) with a 2 cm center-to-center distance were attached along the direction of the muscle fibers on the bellies of the evaluated muscles [17].

Measurements were conducted using a leg extension machine fixed at a 75 degree knee flexion angle. First, the MVC measurement was performed to determine 100% muscle activation. The subject was instructed to press against a stable bar as strongly as possible for 5 s. This measurement was conducted for both the left and right legs. Muscle activation levels of 15%, 30%, and 60% of MVC were determined using MyoResearch XP software (Noraxon USA, Inc., Scottsdale, AZ, USA). Each activation threshold was displayed on a computer screen as a wide line, providing visual biofeedback for the subject. The subject was then given time to familiarize themselves with each threshold by performing a trial on both the right and left limbs. During this familiarization phase, participants completed 3–4 repetitions at each activation level in a self-selected order.

Once the subject was ready for the actual measurements, the visual biofeedback was turned off. A 5 min break was given between the familiarization phase and the main measurement. During the test, the subject was instructed to press against the stable bar for 5 s at a specific percentage of their perceived force level (at 15%, 30%, and 60% of MVC) when the EMG activity was recorded. Each measurement was performed twice for each limb.

#### 2.2.2. Balance Assessment

Balance was assessed using the ALFA stabilometric platform (AC International East, Knurów, Poland). Prior to data collection, the platform was calibrated in accordance with the manufacturer’s instructions to ensure measurement accuracy. During the test, participants were instructed to stand barefoot on the center of the platform in an upright and natural posture, with their arms resting by their sides. They were asked to remain as still as possible, looking straight ahead with their eyes open. Each trial lasted 60 s and was performed once under consistent conditions.

The following six center of pressure (COP)-based variables were extracted from the measurement and analyzed:

- Center of pressure (COP) deviation range in the anterior–posterior (AP) direction (cm) (Length y), measuring the total range of sway forward and backward. A larger value may indicate poorer postural control in the sagittal plane.
- COP deviation range in the medial–lateral (ML) direction (cm) (Length x), measuring the total range of side-to-side sway. It reflects lateral stability and control of hip and trunk muscles.
- COP deviation velocity in the AP direction (cm/s) (Velocity y), representing the speed of postural adjustments forward and backward. A higher velocity can indicate increased postural instability or compensatory movements.
- COP deviation velocity in the ML direction (cm/s) (Velocity x), indicating the speed of side-to-side sway. Like Velocity y, elevated values suggest less control over balance.
- Total path length of the COP (cm) (Total Length). This is the total distance covered by the COP trajectory over time. It is a general indicator of postural stability; longer paths typically reflect reduced balance.
- 95% confidence ellipse area (cm^2^) (Area 95%), representing the area encompassing 95% of the COP data points. A larger area is associated with greater postural sway and instability.

These variables were selected because they represent both the extent and dynamics of postural sway, providing a comprehensive assessment of balance performance. They are commonly used in posturography and have established reliability in evaluating balance impairments in both clinical and research settings.

### 2.3. Data Analysis

The EMG data were collected at a sampling frequency of 1500 Hz and processed according to SENIAM guidelines [16,17,18]. The signal was processed using MATLAB R2025a software (The MathWorks, Natick, MA, USA). To prepare the data for further analysis, the signal was processed using a fourth-order Butterworth bandpass filter with cutoff frequencies ranging from 20 Hz to 500 Hz. For each participant in the study, the initial acquisition involved an isometric task designed to obtain the MVC values for each muscle. The entire signal was normalized by dividing it by the MVC, expressing it as a percentage [%]. The normalized signal was subsequently processed using the root mean square (RMS) method with a window width of 30 samples. With the signal prepared, the mean value of the muscle contraction (MEAN) and the standard deviation (MSD) were then calculated.

### 2.4. Statistical Analysis

Statistical analyses were performed using STATISTICA 13.0 software. Data distribution was assessed with the Shapiro–Wilk test and was found to be normal. The paired *t*-test was used to determine the differences in muscle activity variables between the dominant and non-dominant limbs. An ANOVA with repeated measurements was performed to assess the difference between the muscle activity thresholds of 15%, 30%, and 60%. Pearson’s correlation coefficient (r) was calculated between muscle activity and balance variables. Differences were considered to be statistically significant if the level of the test probability was lower than the assumed level of significance (*p* < 0.05).

## 3. Results

### 3.1. Differences in VMO and VLO Muscle Activity at 15%, 30%, and 60% MVC Thresholds

EMG analysis revealed significant non-linearity between the intended contraction and the actual bioelectrical muscle activity. For both the VLO and VMO muscles, the average EMG amplitude remained below 10% MVC across all thresholds (15%, 30%, and 60%). Significant differences in EMG amplitude were observed between the 15% and 60% MVC thresholds for all muscles on both limbs. However, when comparing 15% vs. 30% MVC with 30% vs. 60% MVC, significant differences appeared only in the following selected variables:

- VLO MEAN (non-dominant side): this was significant at all thresholds (*p* = 0.044 at 15%, *p* = 0.0001 at 30%, and *p* = 0.0001 at 60%).
- VMO MEAN (non-dominant side): significant differences emerged only at 30% and 60% (*p* = 0.0001); it was borderline at 15% (*p* = 0.056).
- VLO and VMO MSD values showed higher variability and only partial significance. For example, VMO MSD (dominant) showed no significant difference at 30% (*p* = 0.152), while other thresholds were significant.

This suggests that muscle activation increased disproportionately, particularly between 15% and 60% MVC, and variability in control was more evident at intermediate thresholds (Table 1).

### 3.2. Differences in VLO and VMO Muscle Activity Between the Dominant and Non-Dominant Sides

No statistically significant differences were found between the dominant and non-dominant sides in either the mean muscle activation or activation error (Table 2 and Table 3).

### 3.3. Correlation Between VMO and VLO Muscle Activity and Balance

The correlation analysis revealed that only one postural control variable, Area 95% (COP sway area), demonstrated a consistent and significant association with the EMG measures. A strong positive correlation was found between non-dominant VLO activity (both MEAN and MSD) and Area 95% at all three thresholds, as follows:

- 15% MVC: MEAN r = 0.63* and MSD r = 0.61*;
- 30% MVC: MEAN r = 0.51* and MSD r = 0.53*;
- 60% MVC: MEAN r = 0.35* and MSD r = 0.39*.

No other balance parameter, such as path length, COP velocity, or COP deviation in AP/ML, showed a significant relationship with VLO/VMO activity. Furthermore, no significant correlations were observed for the dominant limb muscles or for VMO on either side in relation to postural variables. These results indicate that neuromuscular control of the non-dominant VLO muscle may play a critical role in maintaining postural stability, particularly as it relates to the sway area (Table 4).

## 4. Discussion

The most significant finding of this study is that older adults exhibit substantial discrepancies between their intended and actual levels of muscle activation. These results suggest that older adults may experience deficits in perceiving muscle activation, leading to discrepancies between intended and actual muscle engagement. This observation is supported by EMG data showing that, in the absence of visual feedback, participants consistently underactivated their muscles across all target intensities. Despite successfully reaching the required activation levels during the familiarization phase with visual feedback, their muscle activation during experimental trials remained below 10% MVC for all three intended levels (15%, 30%, and 60% MVC). This finding indicates a clear discrepancy between intended and actual activation, and suggests an impaired internal perception of effort or proprioceptive control. Such an underestimation of effort without external cues may reflect age-related deterioration in sensorimotor integration and may contribute to difficulties in daily functional motor tasks, ultimately increasing fall risk.

Studies suggest that skeletal muscle mass and force-generating capacity decline with age, with knee extensor muscles being particularly affected, and that age-related changes in force control are influenced by muscle group specificity, the intensity of the contraction, and habitual physical activity levels [10,19,20]. This decline contributes to mobility limitations and postural instability, increasing the risk of falls [19,20]. Some potential mechanisms underlying these proprioceptive deficits in older adults have been suggested [10,19]. Age-related neuromuscular adaptations, including reduced spindle sensitivity, altered afferent feedback, and diminished central processing efficiency, may impair accurate sensorimotor integration. Additionally, structural and functional changes in the somatosensory cortex, as well as slowed nerve conduction velocity, likely contribute to a degradation in proprioceptive acuity. These neurophysiological changes can lead to imprecise muscle activation control, especially at lower force thresholds where fine motor adjustments are critical [10,19,20].

The control of muscle force is mediated by motor unit recruitment and firing rates, which influence the EMG signal and force output [21]. Studies report that older adults exhibit greater variability in muscles, especially at lower force levels [21,22]. It was also emphasized that this variability may impair their ability to efficiently perform daily activities as weaker muscles require greater effort to generate the necessary force [23].

However, some studies have reported that perception errors decrease when force demands reach 20% MVC, suggesting that very low force levels are inherently difficult to control [2,14]. Franco et al. [2] reported that at higher thresholds, the error decreased, indicating that greater muscle-generated force is easier to control. However, in our study, the error at all MVC thresholds remained similar and did not significantly differ across all three thresholds. Despite prior familiarization with the load magnitude, older adults in our study still struggled with appropriate muscle recruitment. Although a near-linear relationship between muscle force and EMG activity has been reported in the literature [21,22], such a pattern was not observed in our study. This discrepancy may stem from methodological differences, particularly the absence of visual feedback in our protocol, and the reliance on EMG rather than direct force output measures. Previous studies have shown that the task design, including the feedback type and force level, can significantly influence variability and accuracy in older adults’ force control [2,9,14,15].

On the other hand, some authors have suggested that healthy older adults might be capable of generating the required force for mobility tasks. However, optimal performance might be compromised by deficits in muscle function, such as force control, which are particularly evident during submaximal effort [9,15]. Therefore, some researchers have indicated that larger errors occur at higher force levels rather than at lower force thresholds [2,9,15]. It has been reported that the relationship between generated force and knee extensor activation is nearly linear [21,22]. However, other studies indicate that knee extensor strength is a key determinant of functional performance, with a threshold beyond which increased activation—despite recruiting more motor units—does not lead to greater force production [10]. There are some reports indicating that the magnitude of error in recognizing the amount of generated force increases with age [5,24]. Studies have observed that older adults exhibit greater variability than younger individuals across all perceived force level variables, suggesting greater difficulty in controlling the production of a given force level regardless of the intensity of the muscle contraction [1,2]. Recent findings indicate that proprioceptive deficits in older adults are more pronounced at lower force levels, with increased variability in force perception and control, particularly below 2–10% MVC [15], leading to difficulty maintaining intended force levels and limb trajectories. Some researchers have suggested that this may be due to the fact that, during daily activities, muscle activation levels are typically higher than 10% [14,15]. Consequently, muscles are not accustomed to such low activation levels, leading to greater errors in force recognition. Moreover, it was indicated that force perception errors were reduced in older subjects when loads of 20% MVC were applied in comparison with a younger group [13,14]. However, previous studies examining discrepancies between intended and actual muscle activation in older adults have reported mixed findings, which may stem from differences in the study design, such as the use of force output versus EMG as an outcome measure, the inclusion of visual feedback, or variations in the contraction type and intensity. For example, some researchers observed greater variability in force control at low intensities (<10% MVC), particularly when no visual cues were provided [14,15], while others reported increased errors or variability at higher force levels due to limitations in motor unit recruitment or impaired modulation of motor output [2,9,15]. These inconsistencies suggest that force variability in aging is task- and context-dependent, and may be influenced by the type of feedback provided, task familiarity, and muscle group tested. In our study, which relied on EMG-based assessments without visual feedback, we observed consistently low activation regardless of the target threshold, highlighting a potentially unique manifestation of proprioceptive deficits that aligns with studies using similar non-feedback paradigms.

It was also reported that proprioceptive deficits at lower thresholds may translate into balance impairments [3,23]. In our study, we also observed that older adults struggled to accurately perceive force thresholds of varying magnitudes. The fact that they activated muscles to a similar extent regardless of the intended force level suggests that these impairments may also affect balance in daily activities. It has been suggested that proprioceptive deficits at lower thresholds may contribute to balance impairments as greater variability in force perception could make it difficult for older adults to gauge the appropriate force for different tasks [1,16]. Moreover, in our study, a correlation analysis highlighted the importance of the non-dominant VLO muscle in postural stability. Among all balance parameters, only the sway area (Area 95%) showed a consistent and significant relationship with EMG activity. Specifically, a strong positive correlation was found between Area 95% and both the mean and variability of VLO activation on the non-dominant side across all contraction thresholds. In contrast, no significant associations were observed for the dominant limb or for the VMO muscle. These findings suggest that effective control of the non-dominant VLO muscle may be particularly relevant in limiting postural sway, potentially reflecting its compensatory or stabilizing role in balance regulation.

Some researchers suggest that proprioceptive differences between the dominant and non-dominant sides may be influenced by multiple factors [25,26,27,28]. One factor is hemispheric specialization, where the right hemisphere—responsible for controlling the left side of the body—is strongly associated with sensory and spatial processing, potentially leading to better proprioception in the non-dominant limb [25,26]. Another factor is the functional role of each limb; the dominant limb is primarily used for precise and complex motor tasks, whereas the non-dominant limb often plays a stabilizing role, which could affect proprioceptive sensitivity [27]. Additionally, experience and training may contribute to these differences as the dominant limb is more frequently engaged in daily activities, potentially leading to variations in proprioceptive acuity between limbs [28]. In our study, no differences were observed between the dominant and non-dominant sides at any of the thresholds, both in terms of the mean and the activation error. The absence of significant differences between the dominant and non-dominant limbs at any of the tested thresholds may be partly explained by the low level of actual muscle activation observed in the study, which remained below 10% MVC across all conditions. At such low intensities, motor output likely primarily involves low-threshold motor units and does not fully engage the neuromuscular system in a way that would reveal functional asymmetries. It is possible that side-to-side differences in proprioceptive sensitivity or neuromuscular control may only become evident at higher activation levels where greater motor unit recruitment and sensorimotor precision are required. This suggests that future studies should consider including a broader range of intensities to explore potential limb dominance effects more fully.

Some authors have emphasized that, given the decline in proprioception and force control with aging, training programs should focus on enhancing neuromuscular coordination and force steadiness [9]. Proprioceptive training, including balance exercises, resistance training, and motor control drills, has shown promise in improving postural stability [2,9]. Based on the study results, we suggest that exercises emphasizing force differentiation at varying intensities (e.g., 15%, 30%, and 60% MVC) may help older adults develop better control and reduce motor output variability. Implementing targeted neuromuscular training to improve recruitment patterns at different force levels may also enhance balance and potentially reduce fall incidence [29,30,31].

Some limitations should be acknowledged. First, the study sample consisted of physically active older adults, which may limit the generalizability of our findings to more sedentary or frail elderly populations. Individuals with lower physical activity levels may exhibit different proprioceptive and neuromuscular control patterns, potentially leading to greater deficits in muscle activation perception and force control. Future research should include a broader range of participants, including those with varying levels of physical activity and frailty, to better understand the extent of proprioceptive decline in aging populations. The study also focused on isometric contractions at specific muscle activation thresholds but real-life motor tasks often involve dynamic movements and varying force requirements. Future studies should investigate proprioceptive control during dynamic activities, such as gait or postural transitions, to better understand how proprioceptive deficits impact functional mobility and fall risk.

## 5. Conclusions

In conclusion, proprioceptive deficits in older adults may contribute to impaired motor control and decreased postural stability. Our results suggest that older adults experience deficits in muscle activation perception, leading to discrepancies between intended and actual muscle engagement. This may negatively impact functional task performance and increase fall risk. From a clinical perspective, these proprioceptive deficits may have significant implications for daily functional performance. An impaired ability to perceive and regulate muscle activation can compromise postural stability, especially in challenging or unpredictable environments, thus increasing the risk of falls. Our findings support the rationale for targeted interventions that focus on enhancing proprioceptive function, such as sensorimotor training, balance exercises with visual or haptic feedback, and neuromuscular rehabilitation programs designed to restore joint position sense and motor precision. Future studies should consider analyzing a broader range of muscle groups, including postural and deep stabilizing muscles, which play a critical role in balance maintenance. Moreover, incorporating training interventions focused on improving proprioception, neuromuscular coordination, and balance responses may contribute to the development of effective fall-prevention programs for older adults. This approach may offer valuable insights for reducing the incidence of falls and related injuries in this vulnerable population.

## Figures and Tables

**Table 1:** Differences in VMO and VLO muscle activity at 15%, 30%, and 60% MVC thresholds.

Outcome Measure	15% MVC		30% MVC		60% MVC	
	%	*p* *	%	*p* **	%	*p* ***
VLO MEAN ND	5.61	0.044	6.87	0.0001	9.13	0.0001
VLO MEAN D	5.05	0.0001	6.99	0.001	8.52	0.0001
VMO MEAN ND	5.20	0.056	6.23	0.0001	8.69	0.0001
VMO MEAN D	4.55	0.0001	6.50	0.004	8.05	0.0001
VLO MSD ND	2.42	0.260	2.75	0.0001	3.69	0.0001
VLO MSD D	2.11	0.0001	2.95	0.060	3.33	0.0001
VMO MSD ND	2.04	0.003	2.57	0.0001	3.56	0.0001
VMO MSD D	1.93	0.0001	2.91	0.152	3.34	0.0001

*p* *—*p*-value between 15% and 30%; *p* **—*p*-value between 30% and 60%; *p* ***—*p*-value between 15% and 60%. The *p*-value was post hoc of the study group’s main effect. ND—non-dominant side; D—dominant side.

**Table 2:** Differences in VLO muscle activity between the dominant and non-dominant sides.

Outcome Measure	Side	VLO MEAN	*p*	VLO MSD	*p*
**15% MVC**	ND	5.61	0.16	2.42	0.14
	D	4.87		2.07	
**30% MVC**	ND	6.93	0.91	2.78	0.67
	D	6.99		2.89	
**60% MVC**	ND	9.13	0.13	3.69	0.10
	D	8.45		3.32	

ND—non-dominant side; D—dominant side; *p*—*p*-value.

**Table 3:** Differences in VMO muscle activity between the dominant and non-dominant sides.

Outcome Measure	Side	VMO MEAN	*p*	VMO MSD	*p*
**15% MVC**	ND	5.26	0.16	2.05	0.57
	D	4.55		1.93	
**30% MVC**	ND	6.29	0.71	2.60	0.21
	D	6.59		2.91	
**60% MVC**	ND	8.69	0.17	3.55	0.26
	D	8.05		3.34	

ND—non-dominant side; D—dominant side; *p*—*p*-value.

**Table 4:** Correlation between VMO and VLO muscles for activity and balance.

		VLO/VMO MEAN						VLO/VMO MSD					
		15% MVC		30% MVC		60% MVC		15% MVC		30% MVC		60% MVC	
		VLO	VMO	VLO	VMO	VLO	VMO	VLO	VMO	VLO	VMO	VLO	VMO
**Total length**	ND	0.17	0.09	0.18	0.03	0.16	0.06	0.15	0.06	0.18	0.04	0.21	0.06
	D	0.06	0.03	0.06	0.26	0.09	0.11	0.03	0.04	0.03	0.22	0.07	0.08
**Length x**	ND	0.07	0.12	0.02	0.14	0.01	0.19	0.12	0.14	0.02	0.15	0.05	0.12
	D	0.26	0.21	0.20	0.15	0.24	0.19	0.34	0.19	0.28	0.20	0.32	0.20
**Length y**	ND	0.06	0.09	0.11	0.20	0.24	0.21	0.03	0.10	0.08	0.17	0.28	0.16
	D	0.04	0.03	0.13	0.01	0.05	0.07	0.01	0.06	0.01	0.04	0.08	0.10
**Velocity x**	ND	0.20	0.07	0.15	0.03	0.12	0.03	0.18	0.04	0.17	0.01	0.15	0.03
	D	0.01	0.06	0.02	0.03	0.07	0.11	0.01	0.03	0.05	0.26	0.07	0.11
**Velocity y**	ND	0.15	0.09	0.19	0.07	0.19	0.11	0.13	0.07	0.18	0.06	0.25	0.10
	D	0.10	0.09	0.09	0.02	0.09	0.06	0.07	0.10	0.01	0.18	0.06	0.04
**Area 95%**	ND	0.63 *	0.41 *	0.51 *	0.15	0.35 *	0.13	0.61 *	0.39 *	0.53 *	0.15	0.39 *	0.26
	D	0.24	0.10	0.35 *	0.18	0.31	0.04	0.22	0.09	0.25	0.24	0.25	0.22

ND—non-dominant side; D—dominant side. * *p* < 0.05.

## Data Availability

All data generated or analyzed during this study are included in this published article.

## References

[1] Ferlinc A., Fabiani E., Velnar T., Gradisnik L. (2019). The Importance and Role of Proprioception in the Elderly: A Short Review. Mater. Sociomed..

[2] Franco P.G., Santos K.B., Rodacki A.L. (2015). Joint positioning sense, perceived force level and two-point discrimination tests of young and active elderly adults. Braz. J. Phys. Ther..

[3] Kanekar N., Aruin A.S. (2014). The effect of aging on anticipatory postural control. Exp. Brain Res..

[4] Seynnes O., Hue O.A., Garrandes F., Colson S.S., Bernard P.L., Legros P., Fiatarone Singh M.A. (2005). Force steadiness in the lower extremities as an independent predictor of functional performance in older women. J. Aging Phys. Act..

[5] Enoka R.M., Christou E.A., Hunter S.K., Kornatz K.W., Semmler J.G., Taylor A.M. (2003). Mechanisms that contribute to differences in motor performance between young and old adults. J. Electromyogr. Kinesiol..

[6] Montero-Odasso M., van der Velde N., Martin F.C. (2023). World guidelines for falls prevention and management for older adults: A global initiative. Age Ageing.

[7] Rubenstein L.Z. (2006). Falls in older people: Epidemiology, risk factors and strategies for prevention. Age Ageing.

[8] Haentjens P., Magaziner J., Colón-Emeric C.S. (2010). Meta-analysis: Excess mortality after hip fracture among older women and men. Ann. Intern. Med..

[9] Tracy B.L., Enoka R.M. (2002). Older adults are less steady during submaximal isometric contractions with the knee extensor muscles. J. Appl. Physiol..

[10] Fujita E., Kanehisa H., Yoshitake Y., Fukunaga T., Nishizono H. (2011). Association between knee extensor strength and EMG activities during squat movement. Med. Sci. Sports Exerc..

[11] Visser M., Kritchevsky S.B., Goodpaster B.H., Newman A.B., Nevitt M., Stamm E., Harris T.B. (2002). Leg muscle mass and composition in relation to lower extremity performance in men and women aged 70 to 79: The health, aging and body composition study. J. Am. Geriatr. Soc..

[12] Beretta-Piccoli M., Boccia G., Ponti T., Clijsen R., Barbero M., Cescon C. (2018). Relationship between Isometric Muscle Force and Fractal Dimension of Surface Electromyogram. Biomed. Res. Int..

[13] Vaillancourt D.E., Larsson L., Newell K.M. (2002). Time-dependent structure in the discharge rate of human motor units. Clin. Neurophysiol..

[14] Laidlaw D.H., Bilodeau M., Enoka R.M. (2000). Steadiness is reduced and motor unit discharge is more variable in old adults. Muscle Nerve..

[15] Galganski M.E., Fuglevand A.J., Enoka R.M. (1993). Reduced control of motor output in a human hand muscle of elderly subjects during submaximal contractions. J. Neurophysiol..

[16] Merletti R., Parker P. (2004). Electromyography: Physiology, Engineering, and Non-Invasive Applications.

[17] Hermens H.J., Freriks B., Disselhorst-Klug C., Rau G. (2000). Development of recommendations for SEMG sensors and sensor placement procedures. J. Electromyogr. Kinesiol..

[18] Camata T.V., Dantas J.L., Abrao T., Brunetto M.A., Moraes A.C., Altimari L.R. Fourier and wavelet spectral analysis of EMG signals in supramaximal constant load dynamic exercise. Proceedings of the 2010 Annual International Conference of the IEEE Engineering in Medicine and Biology.

[19] Chen X., Qu X. (2019). Age-Related Differences in the Relationships Between Lower-Limb Joint Proprioception and Postural Balance. Hum. Factors.

[20] Allison K.F., Sell T.C., Benjaminse A., Lephart S.M. (2016). Force Sense of the Knee Not Affected by Fatiguing the Knee Extensors and Flexors. J. Sport Rehabil..

[21] Lo Martire R., Gladh K., Westman A., Äng B.O. (2017). Neck Muscle EMG-Force Relationship and Its Reliability During Isometric Contractions. Sports Med. Open.

[22] Ahamed N.U., Sundaraj K., Alqahtani M., Altwijri O., Ali M.A., Islam M.A. (2014). EMG-force relationship during static contraction: Effects on sensor placement locations on biceps brachii muscle. Technol. Health Care.

[23] Wang H., Ji Z., Jiang G., Liu W., Jiao X. (2016). Correlation among proprioception, muscle strength, and balance. J. Phys. Ther. Sci..

[24] Antcliff S., Welvaert M., Witchalls J., Wallwork S.B., Waddington G. (2021). Assessing Proprioception in an Older Population: Reliability of a Protocol Based on Active Movement Extent Discrimination. Percept. Mot. Skills..

[25] Han J., Anson J., Waddington G., Adams R. (2013). Proprioceptive performance of bilateral upper and lower limb joints: Side-general and site-specific effects. Exp. Brain Res..

[26] Strong A., Grip H., Arumugam A., Boraxbekk C.J., Selling J., Häger C.K. (2023). Right hemisphere brain lateralization for knee proprioception among right-limb dominant individuals. Front. Hum. Neurosci..

[27] Galamb K., Szilágyi B., Magyar O.M., Hortobágyi T., Nagatomi R., Váczi M., Négyesi J. (2018). Effects of side-dominance on knee joint proprioceptive target-matching asymmetries. Physiol. Int..

[28] Acosta-Sojo Y., Martin B.J. (2021). Age-related differences in proprioceptive asymmetries. Neurosci. Lett..

[29] Grgic J., Garofolini A., Orazem J., Sabol F., Schoenfeld B.J., Pedisic Z. (2020). Effects of Resistance Training on Muscle Size and Strength in Very Elderly Adults: A Systematic Review and Meta-Analysis of Randomized Controlled Trials. Sports Med..

[30] Reed-Jones R.J., Dorgo S., Hitchings M.K., Bader J.O. (2012). Vision and agility training in community dwelling older adults: Incorporating visual training into programs for fall prevention. Gait Posture.

[31] Chittrakul J., Siviroj P., Sungkarat S., Sapbamrer R. (2020). Multi-System Physical Exercise Intervention for Fall Prevention and Quality of Life in Pre-Frail Older Adults: A Randomized Controlled Trial. Int. J. Environ. Res. Public Health.

